# Leakage of isopropanol from port protectors used in neonatal care—Results from an in vitro study

**DOI:** 10.1371/journal.pone.0235593

**Published:** 2020-07-07

**Authors:** Louise Björkman Hjalmarsson, Jessika Hagberg, Jens Schollin, Andreas Ohlin

**Affiliations:** 1 Department of Paediatrics, Faculty of Medicine and Health, Örebro University Hospital, Örebro University, Örebro, Sweden; 2 Department of Occupational and Environmental Medicine, Faculty of Medicine and Health, Örebro University, Örebro, Sweden; 3 Faculty of Medicine and Health, Örebro University, Örebro, Sweden; University of Florida College of Medicine, UNITED STATES

## Abstract

**Background:**

To decrease contamination of needleless catheter hubs, caps or port protectors impregnated with isopropanol (IPA) have been developed and shown to be superior to other disinfection methods. The safety of the caps has been questioned, as they can be associated with alcohol leakage across the hub membrane.

**Objectives:**

We evaluated the use of IPA caps and the scrub-the-hub method from the safety standpoint of possible alcohol leakage across the hub membrane.

**Methods:**

Circuits imitating an intravenous line were constructed. Circuits with an IPA cap were flushed with sodium chloride after the hub had been exposed to the cap for 1 hour, 24 hours, and 7 days. At the end of each period the fluid was collected and amounts of IPA in it were measured, using gas chromatography. Scrub circuits without IPA caps were also tested and ethanol from these was measured using the same method.

**Results:**

In this in vitro study, IPA was detected in all samples from cap circuits, and ethanol was detected from all scrub circuits. Leakage increased over time in IPA circuits. After 24 hours and 7 days of exposure, the first injection resulted in higher amounts of IPA; thereafter, the levels decreased. The amounts of ethanol measured from the scrub circuits were low.

**Conclusions:**

IPA caps can cause leakage of alcohol across the hub membrane. Leakage increased over time, and a 30 sec drying time was not sufficient to solve the problem. Scrub-the-hub seems safe to use with regard to alcohol leakage.

## Introduction

Catheter-related infections are a major problem in neonatal medicine, and a common mechanism of infection is contamination of the catheter line via the catheter hub [1–3]. With the aim of reducing hub contamination, two different methods for hub sterilization have been developed. The first method is called scrub-the-hub and is widely used in neonatal intensive care today and is recommended by the Centers for Disease Control and Prevention [4]. The method entails a 15–30 sec mechanical scrubbing of the hub membrane. The other method is based on the use of alcohol-impregnated caps or port protectors, which are attached to the hub when it is not in use. This method is time-saving but involves a greater use of disposable products. In recent studies port protectors have been shown to be superior to other methods of disinfection [5–8]. It was therefore concerning when an article in 2015 [9] showed that isopropanol (IPA), which is the active substance in the port protectors, could potentially leak through the hub membrane, meaning that there would be a risk of injecting small amounts of IPA into the patients bloodstream.

Acute neonatal intoxication with IPA is uncommon but serious intoxications have been reported [10, 11]. In IPA intoxication, IPA is metabolized mainly into acetone, which is known to have several adverse effects on the human body [12, 13] such as central nervous suppression, headache, hypotonia and nausea [10, 12, 14]. These signs might be obvious in adult patients but could very well go undetected if a newborn infant was exposed to a low dose IPA intoxication. It is therefore of outmost importance that the hubs used in neonatal intensive care are completely safe and do not allow any leakage of IPA.

Based on the previously mentioned study [9] we decided to test two commercially available alcohol caps together with two hubs commonly used in neonatal intensive care in order to evaluate potential leakage from alcohol caps. For comparison we also evaluated the leakage of alcohol from the scrub-the-hub method.

## Material and methods

**Fig 1:**
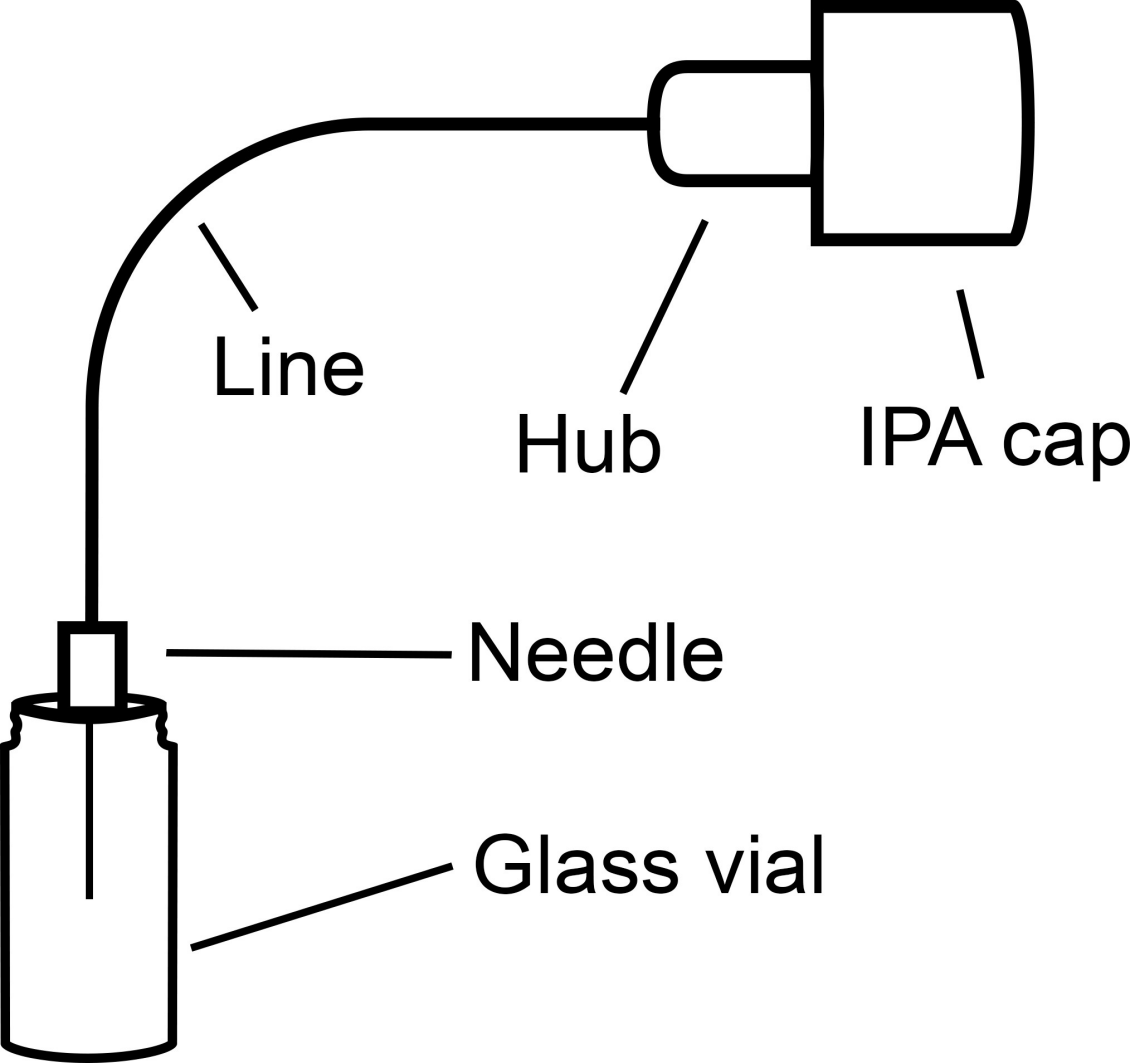
Schematic of IPA cap circuit system.

This study was an in vitro study in a laboratory setting. Two commercially available caps, Curos (Ivera Medial®, Carlsbad, CA, USA) and Swabcap (Braun®, Melsungen, Germany), were tested by creating circuits consisting of an IPA cap, a catheter line measuring 3.9 inches (3-way stopcock + extension line, Mediplast®, Malmö, Sweden) a hub, either Swan-lock (Codan®, Lensahn, Germany) or Bionector (Vygon®, Ecouen, France), a needle (a 0.4 mm × 19 mm, BD Eclipse, Franklin Lakes, NJ, USA) and a 2 ml glass vial (Agilent Technologies®, Santa Clara, CA, USA) (Fig 1).

Four different combinations of circuits were constructed (the Swan-lock hub with Swabcap, the Swan-lock hub with Curos cap, the Bionector hub with Swabcap and the Bionector cap with Curos). Six circuits of each combination were created, for a total of 24 circuits with alcohol caps.

For comparison, two circuits without IPA caps were created using the same types of hubs (Swan-lock and Bionector), and these hubs were instead disinfected using the scrub-the-hub method. Six circuits containing the Swan-lock hub and six circuits containing the Bionector hub were created, for a total of 12 circuits without alcohol caps.

In all circuits the catheter line was prefilled with 0.5 ml sodium chloride containing 0.05% 1-propanol (99% Alfa Aesar®, ThermoFisher GmbH, Karlsruhe, Germany). The 1-propanol was added to the sodium chloride as an internal standard (IS) to enable detection of any evaporation losses during the experiment and thereby improve the precision and accuracy of the method.

**Fig 2:**
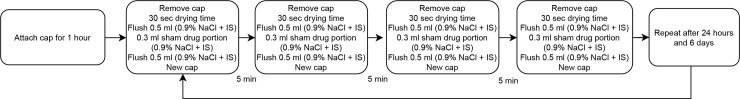
Injection scheme for IPA circuits. IS = Internal standard.

All cap circuits were tested according to the scheme shown in Fig 2. After the cap had been removed, the hub membrane was left to dry for 30 seconds, according to recommendations from the cap manufacturers. Each injection was divided into three portions to imitate the process of a drug administration. The syringe (LUER LOCK 2 ml, Codan®, Rodby, Denmark) was removed from the hub and reattached before each injection. Circuits with an IPA cap were flushed with sodium chloride after the hub had been exposed to the cap for 1 hour, 24 hours, and 7 days. The fluid was collected in a separate 2 ml vial and immediately sealed with a 9 mm blue screw cap with PTFE septa (Agilent Technologies®, Santa Clara, CA, USA).

**Fig 3:**
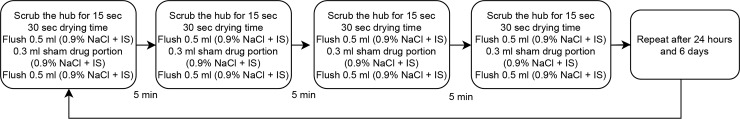
Injection scheme for scrub circuits. IS = Internal standard.

The scrub circuits were disinfected by scrubbing the hub membrane with a wipe soaked in chlorhexidine 5 mg/ml (Fresenius Kabi®, Uppsala, Sweden) for 15 seconds, after which the membrane was allowed to dry for 30 seconds; then the scrub circuits followed the same injection scheme as shown in Fig 3 with flushing of the circuits after 1 hour, 24 hours, and 7 days.

The amounts of IPA and ethanol were measured by gas chromatography coupled to a flame ionization detector (GC-FID). In summary, a splitless injection was used to inject 1 μl of the sham drug injection into a 60 m (0.25 mm id, 1.00 μm) DB-1 column (J&W Scientific; Folsom, CA, USA). The injector temperature was 200°C. The oven temperature program was as follows: initial temperature of 65°C (held for 5.0 minutes), then the oven temperature was increased by 80°C/min to 180°C with a final hold of 2.5 min. Calibration curves were adopted from eight calibration solutions of IPA (99.6%; Acros Organics, Geel, Belgium) and ethanol (99.5%; Kemetyl AB, Haninge, Sweden) in the concentration range of 1–2000 μg/ml. All calibration solutions were prepared in 0.9% sodium chloride solution containing the same concentration of IS as the sham drug injections. The amounts of IPA and ethanol were calculated in μg/injection. The gas chromatography analysis was initiated within 2 hours of the injections being done.

To enable gas chromatography analysis the same day as the injections and to not risk evaporation from the vials, the experiments took place within two weeks, with half of the circuits tested each week.

### Statistical analysis

The amounts of IPA and ethanol in each vial were measured in μg. Median and maximum leakage per circuit was calculated in μg using Excel. Based on previous intoxications [10, 11, 15–17] and previous studies [9], alcohol leakage >1130 μg was defined as the critical amount per injection.

## Results

**Fig 4:**
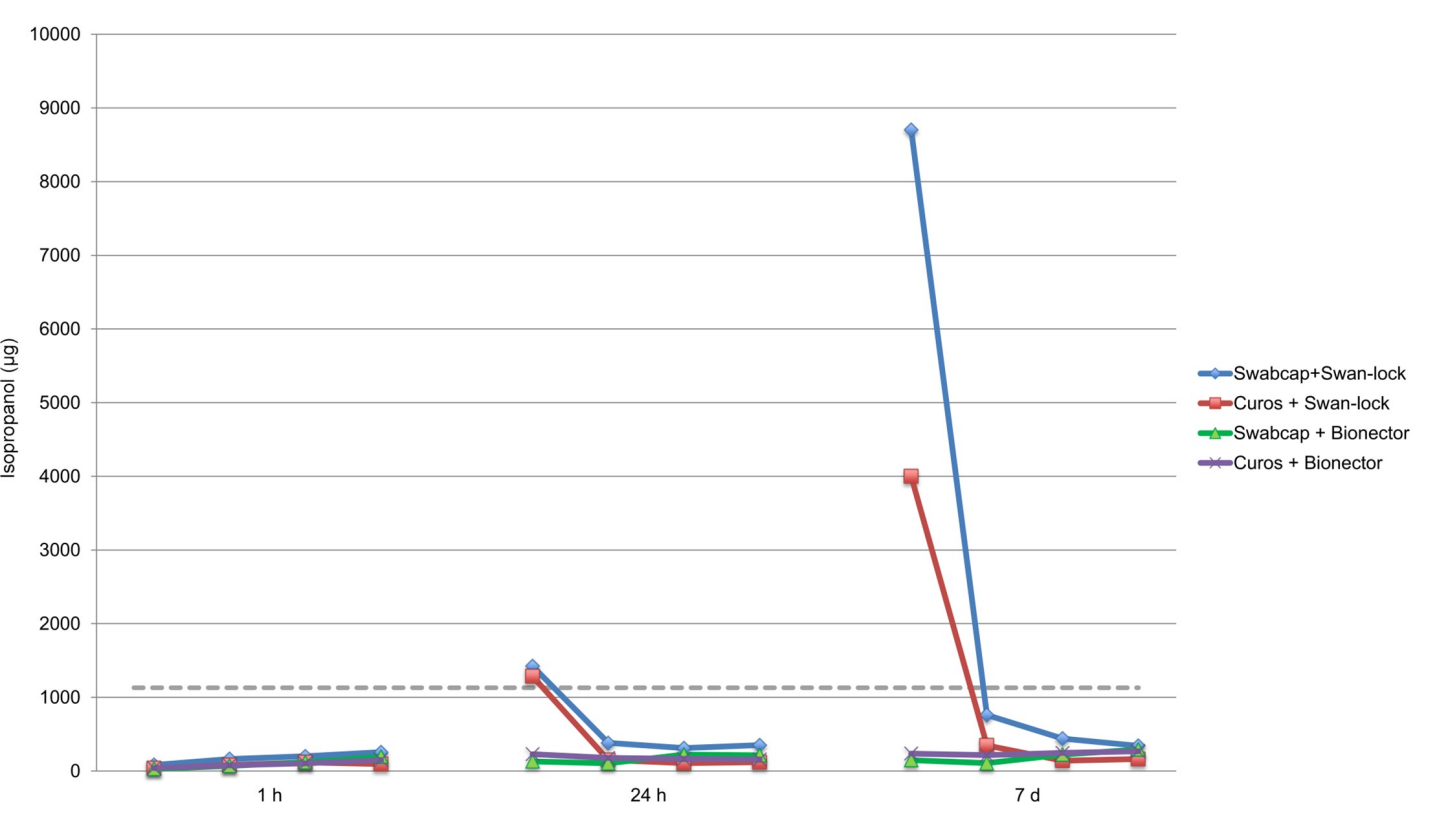
IPA leakage. Median amounts of isopropanol (IPA) derived from measurements of six replicates of each cap circuit. Circuits with the same cap and hub combination are represented by the same color. The four dots per line illustrate the median value of each of the four consecutive injections administered to the six circuits. The black dotted line represents the critical concentration of 1130 μg.

IPA could be detected in all samples from circuits with alcohol caps. In addition, ethanol could be detected in all samples from circuits that had undergone the scrub-the-hub method. Median and maximum leakage per circuit type is presented in Table 1. The median amount of IPA recorded from each injection (i.e. four replicates) and circuit type is presented in Fig 4. Alcohol leakage increased over time in the cap circuits but this trend was not observed in the scrub circuits.

**Table 1:** Median and maximum leakage per circuit type. Median amount of isopropanol or ethanol per circuit type after 1 hour, 24 hours, and 7 days of cap exposure. Each value presented in the table is a median value of 24 injections administered per circuit type. Maximum amount of isopropanol or ethanol recorded in one injection per circuit type. Amounts >1130 μg are marked in bold.

	Median amount of IPA/ethanol per circuit type in μg			Maximum amount of IPA/ethanol in one injection in μg	
**Exposure time**	1 h	24 h	7 d	24 h	7 d
**Swan-lock + Swabcap**	162	405	550	**1755**	**9850**
**Swan-lock + Curos**	72	166	218	**1363**	**5024**
**Bionector + Swabcap**	92	154	200	372	482
**Bionector + Curos**	72	175	235	**1550**	497
**Swan-lock + Scrub**	4	10	6	85	17
**Bionector + Scrub**	7	15	25	66	73

When the circuits had been exposed to IPA caps for one hour, the IPA levels were below the critical amount for all circuits. After 24 hours of alcohol cap exposure, higher amounts of IPA were detected. In the circuits containing Swan-lock hubs, critical amounts (>1130 μg) were reached in the first injection, and thereafter the levels were lower for the following three injections and did not reach the critical cut-off value. After 24 hours of cap exposure, one of the Bionector circuits contained higher IPA amounts than the other identical circuits.

The same pattern could be observed when the circuits had been exposed to an alcohol cap for a total of 7 days. The first injection reached critical amounts for all Swan-lock circuits. In the Bionector circuits the IPA amounts did not exceed the critical cut-off in any injection.

**Fig 5:**
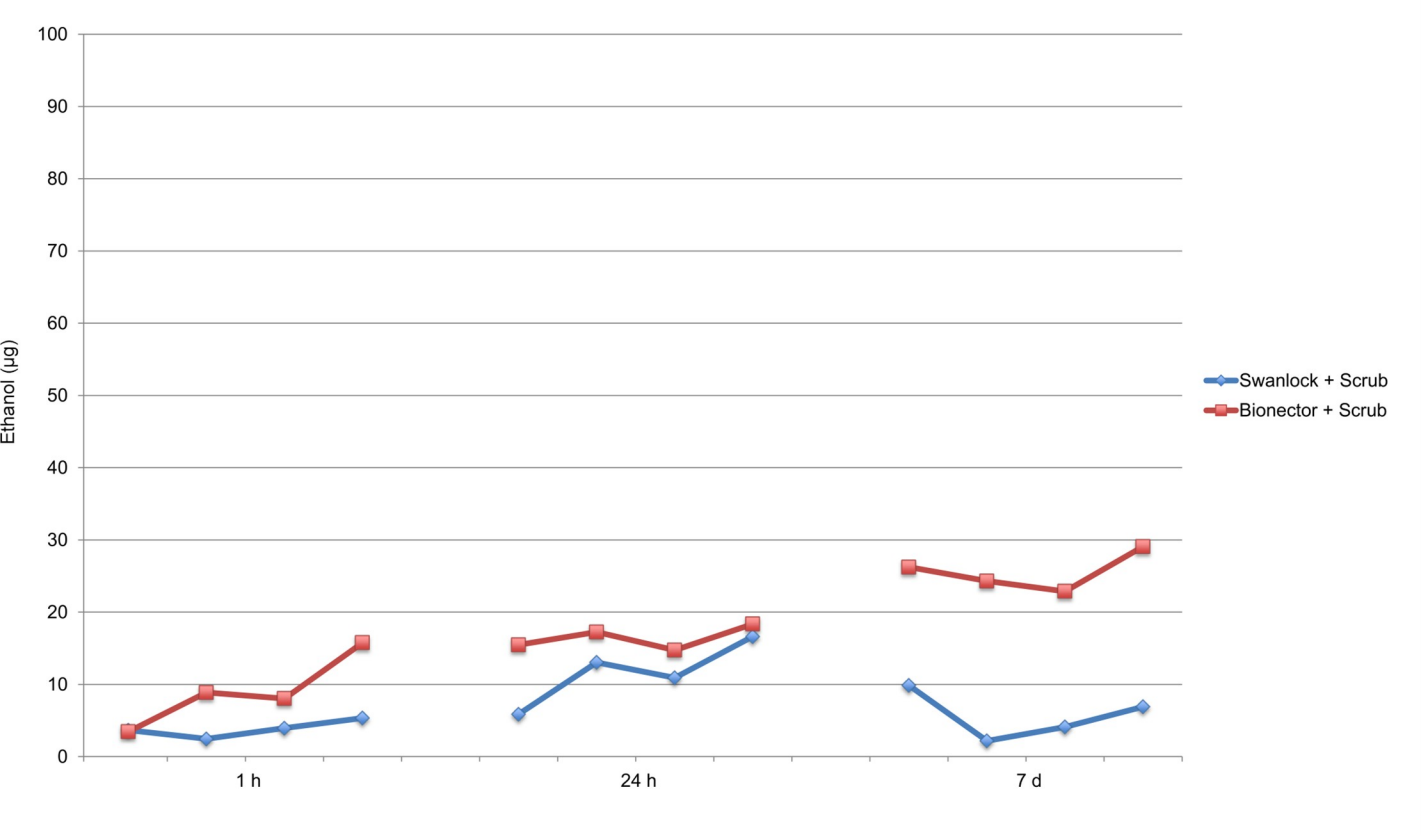
Ethanol leakage. Median amounts of ethanol derived from measurements of six replicates of each scrub circuit. The circuits with the same hub type are represented by the same color. The four dots per line illustrate a median value of each injection administered to the six circuits.

The amounts of ethanol that had passed across the membrane in the scrub circuits are presented in Fig 5. For the Swan-lock circuits, the maximum leakage was 85 μg and for the Bionector circuits the maximum leakage was 73 μg.

The sodium chloride with IS, which was used for the injections, was analyzed each day for ethanol and IPA and did not contain any alcohol except for IS.

## Discussion

The main result of this in vitro study is that we can confirm the results from Sauron et al that there is a risk of IPA leakage when IPA caps are used [9]. This is troublesome as alcohol caps have been shown to be effective and are considered to be an important tool against catheter-associated infections [5–8]. The largest IPA leakage in our study was detected when the hub had been exposed to an alcohol cap for 7 days. This suggests continuous leakage of alcohol over time from the cap into the hub, but this is only a speculation, as this experiment was not designed to show how the IPA passed across the hub membrane. This study clearly shows that different combinations of caps and hubs resulted in various degrees of IPA leakage. Since there are numerous hubs and caps on the market, it would therefore be desirable to have recommendations regarding which combinations are safe to use and which should be avoided.

In our study there was leakage in the Bionector circuits, although not reaching the critical concentration except for one injection in one of the circuits, suggesting that Bionector is a safer choice than Swan-lock when used together with IPA caps.

In the scrub-the-hub circuits (without IPA caps) ethanol could be detected, but the levels were low compared with the IPA concentrations in the capped circuits, and the scrub circuits did not show the same trend of increased concentrations over time. Therefore, scrubbing the hub seems to be a safe method to use with regard to alcohol leakage. One should also keep in mind that ethanol is a less toxic alcohol than IPA, and to improve the safety profile of alcohol caps, it might be possible to exchange IPA for ethanol in the caps, thus decreasing the risk of toxicity.

In this study, the IPA leakage was lower than previously shown. This can probably be explained by the 30 seconds of drying used in this study, allowing less residual alcohol on the membrane to be flushed into the catheter line compared to when no drying time was allowed [9].

One of the difficulties with IPA is that the exact toxicity of the compound is unknown. Previous reports of IPA intoxications indicate that measured toxic blood concentrations seem to vary between 25 and 520 mg/dl, which is a twenty-fold variation [10, 11, 15–17]. This would translate into 1130–7700 μg as the critical amount per injection. We decided to use 1130 μg as our threshold, partly because this was the level that Sauron et al. [9] used, but also because of the fact that IPA caps are designed to be used on central lines for patients in intensive care. These patients often have multiple lines and receive a high number of injections per day, which increases the risk of IPA accumulation. This risk becomes extra evident in neonatal care, where the levels might constitute a significant part of the circulation blood volume.

A limitation of the study is that it is relatively small, and only four combinations of caps and hubs were tested. We can therefore not know whether another cap and hub combination might cause alcohol leakage or be free from leakage issues. Another limitation is the in vitro setting; if a future study could be performed in an animal model, a more correct measurement of alcohol levels could be made.

The consistency of the results strengthens the study, meaning that circuits with the same cap and hub combination showed similar results, and therefore, it seems unlikely that a larger sample size would change the conclusions. Moreover, this study showed similar results to the study by Sauron et al. [9].

We conclude that there is a risk of IPA leakage when IPA-impregnated caps are used. This leakage increased the longer the hubs were exposed to an IPA cap, and a 30 second drying time was not sufficient to solve the problem. There is reason to question the safety of these products with regard to risk of alcohol leakage. Scrub-the-hub seems to be a safe method to use concerning risk of alcohol leakage.

## Supporting information

S1 Data (XLSM)

S2 Data (XLSX)

## Abbreviations

IS: Internal standard
IPA: Isopropanol
